# The clinical significance of *BRAFV600E* mutations in pediatric papillary thyroid carcinomas

**DOI:** 10.1038/s41598-022-16207-1

**Published:** 2022-07-25

**Authors:** Yangsen Li, Yuanyuan Wang, Liwen Li, Xinguang Qiu

**Affiliations:** Department of Thyroid Surgery, The First Affiliated Hospital of Zhengzhou University, Zhengzhou, 450052 China

**Keywords:** Cancer, Genetics, Diseases, Health care, Oncology, Risk factors, Signs and symptoms

## Abstract

This study aimed to review the clinical significance of BRAFV600E mutations in pediatric papillary thyroid carcinoma (PTC). From 2018 to 2021, 392 pediatric thyroid operations were performed in the first affiliated Hospital of Zhengzhou University. Of these, 169 patients underwent their first operation in our hospital and were histopathologically diagnosed as papillary thyroid carcinoma. BRAFV600E gene mutation detection was performed in these 169 pediatric patients to investigate the correlation between BRAF gene mutations and clinicopathological features. Ninety-seven of our 169 patients had a BRAFV600E mutation, with a mutation rate of 57.4%. The incidence of BRAFV600E was higher in boys than in girls, and in the 13–18-year age group as compared with the 6–12-year age group (P < 0.05). The positivity rate of BRAFV600E in unilateral PTC (67.7%) was significantly higher than the ones in bilateral PTC (28.9%). The occurrence of diffuse microcalcification of the thyroid negatively correlated with the presence of BRAFV600E mutations. BRAFV600E mutations were found more frequently in patients with smaller tumor size, a lack of multifocality, lower TSH levels and central lymph node metastasis. During the follow-up time, 70 patients were treated with iodine-131. Eight patients required a second surgery (All had cervical lymph node recurrence). BRAFV600E mutations do not suggest a more aggressive course in papillary thyroid carcinoma in pediatric patients in the short term.

## Introduction

The incidence of thyroid cancer in pediatric patients is significantly lower compare to adults, and there are significant differences between pediatric patients and adults in molecular pathology as well as in clinical manifestations^1^. Pediatric differentiated thyroid carcinomas include papillary thyroid carcinoma (PTC) and follicular thyroid carcinoma (FTC), of which PTC is the more common type. Unlike adult patients, PTC in pediatric patients is often asymptomatic, has a slow rate of growth and is hence more likely to present with advanced disease^2^. Children with PTC who present with multifocal, aggressive disease with invasion of blood vessels, the recurrent laryngeal nerve, trachea and esophagus have a poor prognosis^3^. Statistics show that the annual incidence of thyroid cancer in children and adolescents in China is approximately 0.44/100,000, and the mortality rate is 0.02/100,000^4^. Globally it has been found that the annual prevalence of thyroid cancer in adolescents is increasing^5,6^. Therefore, early diagnosis of PTC is the key to improving its prognosis. It is well known that BRAFV600E mutation rates are high in adult thyroid cancer^7,8^. However, the use of BRAFV600E as a molecular marker in adolescent thyroid cancer remains controversial^9–11^. This might be due to the limited number of reported cases. In this study, we aimed to evaluate the correlation between BRAFV600E mutations and their clinicopathological significance in pediatric PTC cases.169 cases of pediatric PTC in which we studied is the largest number of cases reported so far. This study would be an interest to all whom dealing with pediatric thyroid cancer.

## Patients and methods

### Patients

The study protocol was approved by the first affiliated Hospital of Zhengzhou University ethics and scientific review board (2019-KY-0202). Written informed consent were obtained from all the childre’s legal guardians, and the participants of age 16 to 18 provided their own informed consent to participate in the study. We confirm that all methods were performed in accordance with the relevant guidelines and regulations. We included 169 children (40 males and 129 females), ranging in age from 6 to 18 years, who had received their first surgical treatment in our hospital and in whom postoperative histopathology showed papillary thyroid carcinoma. There was no history of radiation exposure in these patients. The following data were recorded in each patient: age, sex, tumor size, unilateral/bilateral involvement, presence/absence of diffuse microcalcification and multifocal, extrathyroidal extension, Tumor Nodes Metastasis (TNM) stage, Thyroid-Stimulating Hormone (TSH) level, treatment received, and findings on follow-up. All cases were diagnosed and managed in accordance with the 2015 American Thyroid Association (ATA) guidelines for the diagnosis and treatment of pediatric thyroid nodules and differentiated thyroid cancer^12^. All histologic slides were reviewed by two independent pathologists according to the 2010 American Joint Committee on Cancer (AJCC) Guidelines, Edition 7.

### DNA extraction

The mutational analysis was performed on formalin-fixed, paraffin-embedded tumor tissue from the thyroid resection specimens. Three to six pieces of unstained section (10 μm thick) from the tumor were deparaffinized and macro-dissected. DNA was extracted using a Qiagen tissue DNA extraction kit according to the manufacturer’s protocol (QIAamp DNA FFPE Tissue Kit, QIAGEN, Germany).

### Gene detection

BRAF exon 15 was amplified using a BRAF mutation detection kit. Briefly, 5 μL DNA was added to 35 μL of the amplification system. The upstream primer sequence of BRAF was 5′-ATGCTTGCTCTGATAGGAA-3′ while the downstream primer sequence was 5′-GCATCTCAGGGCCAAA-3′. Polymerase Chain Reaction (PCR) amplification was then performed. The amplification conditions were as follows: pre-denaturation at 95 °C for 15 min; then denaturation at 94 °C for 30 s; annealing at 54 °C for 20 s; extension at 72 °C for 30 s for 35 cycles, the last 72 °C extension being for 10 min. BRAF amplification products were purified and sequenced by the BigDye^®^ Terminator v3.1 Cycle Sequencing Kit (Applied Biosystems, USA). Each sample was tested at least three times to ensure the repeatability of sequencing results.

### Statistical analysis

The Chi-square test was used to assess whether BRAFV600E mutations were associated with age, sex, unilateral/bilateral involvement, presence/absence of diffuse microcalcification and extrathyroidal extension, multifocal/unifocal involvement, TNM stage, TSH level, treatment, and findings on short-term follow-up. Student’s t test and Mann–Whitney test were used appropriately for continuous numerical data, after applying the Shapiro–Wilk test for normality. Continuous data were summarized as median and range or mean ± standard deviation. All statistical calculations were performed using the SPSS Statistics 22.0 (IBM Inc., Armonk, New York, USA). A P value of < 0.05 was considered statistically significant.

## Results

**Figure 1:**
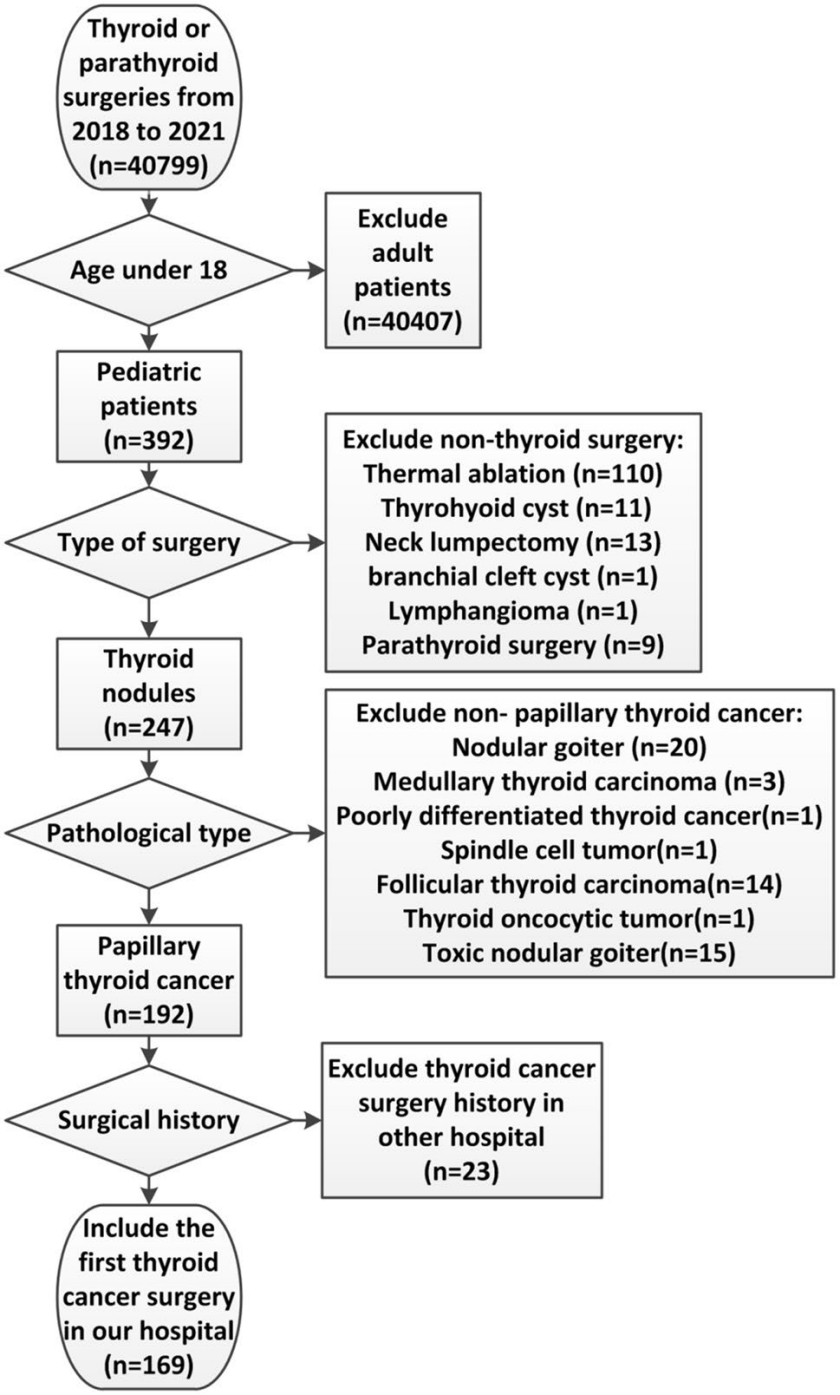
The flow diagram for the inclusion of patients in this study.

From 2018 to 2021, 40,799 thyroid operations were performed in the First Affiliated Hospital of Zhengzhou University. Of these, there were 392 patients aged 0–18 years of which 169 patients were diagnosed as PTC and underwent their first surgery at our hospital. The flow diagram for the inclusion of patients in this study is given in Fig. 1.

In the Table 1, 97 of our 169 patients had a BRAFV600E mutation, with a mutation rate of 57.4%. The rate of BRAFV600E mutation in males (72.5%) was higher than that in females (52.7%) and this difference was statistically significant (P = 0.027). The BRAFV600E mutation rate was higher in children aged 13–18 years as compared with those aged 6–12 years (P < 0.05). Similarly, the mutation rate of BRAFV600E in children with unilateral papillary thyroid carcinoma was significantly higher than that in children with bilateral papillary thyroid carcinoma (P < 0.05). BRAFV600E mutation rate of children with diffuse microcalcification on ultrasonography was significantly lower than that of children without diffuse microcalcification (P < 0.05). For tumor size, the maximum tumor diameter of children with BRAFV600E mutations was smaller than that of those without the mutation. The mutation rate of BRAFV600E in children with multifocal involvement was lower than that in those with unifocal involvement. There was no significant difference in the rate of BRAFV600E mutation in patients with or without extrathyroidal extension. The TSH values in those with BRAFV600E mutations were significantly lower than those without and this difference was statistically significant (P < 0.05). In terms of TNM staging, 45 cases with central node metastasis (72.6%) had BRAFV600E mutations, whereas 38 cases with lateral neck node metastasis (43.7%) had BRAFV600E mutations and this difference was statistically significant. There were five cases of lung metastasis, two of whom had BRAFV600E mutations (40%). Of the remaining 164 patients without lung metastasis, 95 had BRAFV600E mutations (57.9%). The presence of BRAFV600E mutations correlated with central node metastasis, and this correlation was statistically significant. Hence, BRAFV600E mutations did not suggest a more aggressive behavior of papillary thyroid carcinoma in pediatric patients.

**Table 1:** Relationship between BRAFV600E gene mutations and clinicopathological characteristics of papillary thyroid carcinoma in pediatric patients.

Variable	All n = 169	BRAFV600E (+) n = 97	BRAFV600E (−) n = 72	P-value
**Sex**				0.027^a^
Female	129	68 (52.7%)	61 (47.3%)	
Male	40	29 (72.5%)	11 (27.5%)	
**Age (years)**				< 0.05^a^
6 to 12	20	4 (20.0%)	16 (80.0%)	
13 to 18	149	93 (62.4%)	56 (37.6%)	
**Nodule location**				< 0.05^a^
Unilateral	124	84 (67.7%)	40 (32.3%)	
Bilateral	45	13 (28.9%)	32 (71.1%)	
**Diffuse microcalcification**				< 0.05^a^
Yes	27	1 (3.7%)	26 (96.3%)	
No	142	96 (67.6%)	46 (32.4%)	
Nodule size in mm (mean ± SD)	19.2 (11, 32)	15.5 (10, 32)	24.9 ± 11.8	0.008^b^
TSH level in mIU/L (mean ± SD)	2.57 ± 3.1	2.43 ± 2.34	3.10 ± 3.91	< 0.05^c^
**Multifocality**				< 0.05^a^
Yes	59	23 (39.0%)	36 (61.0%)	
No	110	74 (67.3%)	36 (32.7%)	
**Extrathyroidal extension**				0.109^a^
Yes	50	24 (48%)	26 (52%)	
No	119	73 (61.3%)	46 (38.7%)	
**T stage**				< 0.05^a^
T1	66	54 (81.8%)	12 (18.2%)	
T2	34	16 (47.1%)	18 (52.9%)	
T3	60	24 (40.0%)	36 (60.0%)	
T4	9	3 (33.3%)	6 (66.7%)	
**N stage**				0.225^a^
N0	20	14 (70%)	6 (30%)	
N1	149	83 (55.7%)	66 (44.3%)	
**N1 stage**				< 0.05^a^
N1a	62	45 (72.6%)	17 (27.4%)	
N1b	87	38 (43.7%)	49 (56.3%)	
**M stage**				0.652^a^
M0	164	95 (57.9%)	69 (42.1%)	
M1	5	2 (40%)	3 (60%)	

^a^Chi-square test.

^b^Mann–Whitney test.

^c^Independent-samples T test.

**Figure 2:**
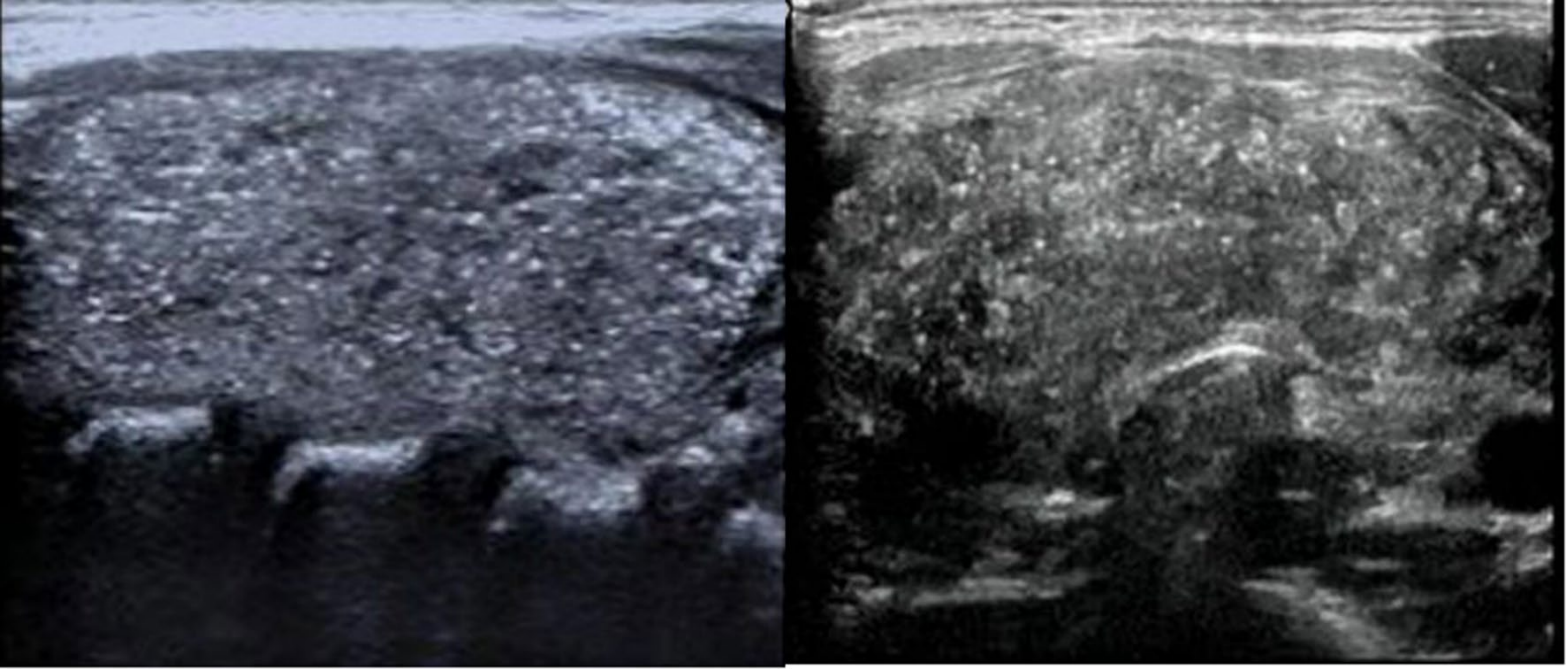
Ultrasonographic images of thyroid in pediatric PTC patients, showing diffuse microcalcification.

In the Table 2, we studied the association between diffuse microcalcification (which is an image showing in Fig. 2) and extra-thyroidal extension, and TNM staging. There was no signifcant difference in the number of patients with extrathyroidal extension with or without diffuse microcalcification. However, we found that diffuse microcalcification has different incidence in different T stages, which was statistically significant.And diffuse microcalcification correlated positively with lateral neck node metastasis and this was correlation statistically significant.

**Table 2:** The relationship between diffuse microcalcification and extra-thyroid extension and postoperative TNM staging.

Variable		All n = 169	Diffuse microcalcification (+) n = 27	Diffuse microcalcification (−) n = 142	P-value
**Extra-thyroid extension**					0.065^a^
Yes		50	12 (24.0%)	38 (76.0%)	
No		119	15 (12.6%)	104 (87.4%)	
**T stage**					< 0.05^a^
T1		66	0 (0%)	66 (100%)	
T2		34	2 (5.9%)	32 (94.1%)	
T3		60	21 (35.0%)	39 (65.0%)	
T4		9	4 (44.4%)	5 (55.6%)	
**N stage**					0.185^a^
N0		20	0 (0%)	6 (100%)	
N1		149	27 (18.1%)	66 (81.9%)	
**N1 stage**					< 0.05^a^
N1a		62	25 (0%)	17 (100%)	
N1b		87	2 (31.0%)	49 (67.0%)	
**M stage**					0.181^a^
M0		164	25 (15.2%)	139 (84.8%)	
M1		5	2 (40%)	3 (60%)	

^a^Chi-square test.

Until the date of publication, the retrospective study of the outcomes in 169 cases follow-up was conducted, patients were followed up for at least 1 month (19 (10, 31) months, range 1–48 months). (Table 3). It was found that 41 cases (58.6%) without BRAFV600E mutations required treatment with iodine-131 as compared with 29 cases (41.4%) with BRAFV600E mutations, and this difference was statistically significant. Eight patients required a second surgery. All had cervical lymph node recurrence. Only two of these eight patients (25%) had BRAFV600E mutations.

**Table 3:** Outcomes of 169 pediatric patients with PTC.

Variable	All n = 169	BRAFV600E (+) n = 97	BRAFV600E (−) n = 72	P-value
**Iodine treatment**				< 0.05^a^
Yes	70	29 (41.4%)	41 (58.6%)	
No	99	68 (68.7%)	31 (31.3%)	
**Second operation**				0.074^a^
Yes	8	2 (25.0%)	6 (75.0%)	
No	161	95 (59.0%)	66 (41.0%)	

^a^Chi-square test.

## Discussion

Most studies on the correlation between BRAFV600E mutations and PTC have focused on adult patients. The frequency of BRAFV600E mutations in adults with PTC has been reported to be between 36 and 83%^13–16^. A recent meta-analysis in adults reported that BRAF mutations are positively associated with tumor aggressiveness^14,17^. In recent years, the incidence of PTC in pediatric patients has been found to be increasing. As compared with adult thyroid cancer, lymph node metastasis is more likely to occur and is more aggressive in pediatric thyroid cancer^13^. However, the prognosis is relatively good^18,19^. The mechanism underlying these differences is unclear. The role of BRAFV600E mutations in adult thyroid cancer is well established, and researchers have now begun to focus on BRAFV600E mutations in pediatric PTC. The incidence of BRAFV600E in pediatric thyroid cancers has been varied^13,20,21^. In our study, of 169 children with PTC, the mutation rate of BRAFV600E was 57.4%, which was different from previous reports^15–23^. This may be related to the large number of patients in our study.

We found that the incidence of BRAFV600E mutations in pediatric thyroid cancer was higher in the age group of 13–18 years. We also found that the rate of BRAFV600E mutations in males (72.5%) was higher than that in females (52.7%). Some studies have not found any correlation of BRAFV600E mutations with gender^24^. These differences may be related to race, region and sample size. The maximum tumor diameter of pediatric patients with BRAFV600E mutations was less than that of patients without BRAFV600E mutations. The mutation rate of BRAFV600E in patients with multifocal disease was lower than that in those with non-multifocal disease. There was no difference in the rate of BRAFV600E mutations in those with extra-glandular invasion as compared with those without, and this was consistent with previous studies^25^. This is in contrast to reports in adults with PTC where the presence of BRAFV600E mutations correlated significantly with extra-glandular invasion, tumor multifocality and tumor size^14,15,17,26,27^. BRAFV600E mutations were found in 45 patients (72.6%) with central lymph node metastasis and 38 cases (43.7%) of children with cervical lymph node metastasis, which was consistent with the findings of Japanese workers^24,28^. BRAFV600E positive rate was not significantly associated with distant metastasis in pediatric PTC, unlike studies in adult PTC^27^.

We maybe the first to study the relationship between diffuse microcalcification and extra-thyroidal extension, and TNM staging.In this study we found that the diffuse microcalcification correlated positively with lateral neck node metastasis and this was correlation statistically significant, which may be considered as a reference for preoperative prediction of lymph node metastasis.

Seventy children were treated with iodine-131. Of these, 29 (41.4%) had BRAFV600E mutations. Of the 99 patients not treated with iodine-13, BRAFV600E mutations were seen in 68.7%. Studies have shown that BRAFV600E mutations in adult PTC may be a predictor for the efficacy of iodine-131^29^. On follow-up of our patients, we found that eight patients with cervical lymph node recurrence underwent a second surgery. Only two of these eight patients had a BRAFV600E mutation. However, in adult PTC, BRAFV600E mutations were strongly associated with recurrence. The presence of a BRAFV600E mutation in adult patients was found to be an independent risk factor and was the single most important factor for prediction of recurrence and long-term prognosis. We were not able to study the prognostic value of BRAFV600E mutations in pediatric PTC because of the short period of follow-up in our patients. It is possible that the pathogenesis and mechanism of pediatric PTC are different from those of adult PTC and the role of BRAFV600E mutations may also be different. Our study analyzed the significance of diffuse micro-calcification in pediatric PTC for the first time. We found that it was positively correlated with cervical lymph node metastasis and this finding requires further study.

In conclusion, our study reports the largest number of pediatric PTC patients till date. We found the mutation rate of BRAFV600E in papillary thyroid carcinoma in pediatric patients to be lower than that in adults. BRAFV600E mutations do not seem to suggest a more aggressive course of papillary thyroid carcinoma in pediatric patients in the short term. Long-term follow-up of our patients is required to study the effect of BRAFV600E mutations on recurrence and survival. It may also be worthwhile to search for other molecular markers in pediatric PTC.

## Data Availability

The datasets used and/or analysed during the current study available from the corresponding author on reasonable request.
